# Disseminated tungiasis in a 78-year-old woman from Tanzania: a case report

**DOI:** 10.1186/s13256-016-1146-6

**Published:** 2016-12-20

**Authors:** Pedro Pallangyo, Paulina Nicholaus

**Affiliations:** Unit of Research, Jakaya Kikwete Cardiac Institute, PO Box 65141, Dar es Salaam, Tanzania

**Keywords:** Tungiasis, Sandfly, *Tunga penetrans*, *Tunga* infestation, Neglected tropical diseases, Case report

## Abstract

**Background:**

Tungiasis is one of the neglected tropical diseases; it affects up to 40% of individuals living in societies with poor housing and sanitation standards. In endemic areas, *Tunga* infestation, which predominantly affects the periungual areas of the lower limbs in humans, is associated with considerable morbidity and poor quality of life.

**Case presentation:**

A 78-year-old woman of African descent presented with pain, inflammation, suppuration, ulceration, and deformation of digits of all four limbs. She had a total of 1146 embedded sand fleas: 812 in lower limbs and 334 in her hands. She was febrile; her full blood count revealed pancytopenia and blood cultures were positive for *Staphylococcus aureus* and *Streptococcus pyogenes* isolates. Furthermore, she had severe hyponatremia. We applied 20% salicylated petroleum jelly followed by the manual removal of embedded sand fleas with a sterile needle. Intravenously administered piperacillin-tazobactam, topical ivermectin, ferrous sulfate, folic acid, tolvaptan, albendazole, multivitamins, and tetanus prophylaxis were instituted. She was discharged home after 16 days of hospitalization.

**Conclusions:**

Tungiasis is a neglected disease of concern in underprivileged societies that is preventable and curable. Early recognition and prompt treatment is crucial to prevent complications in this disease which may potentially mimic other conditions resulting in erroneous management.

## Background

Tungiasis is an infestation resulting from a permanent penetration of the female sand flea (*Tunga penetrans*) into the skin [1]. This cutaneous ectoparasitic infection is one of the neglected tropical diseases that are endemic in marginalized populations, including Sub-Saharan Africa [1–3]. Owing to the flea’s limited flying ability, tungiasis predominantly affects the periungual areas of the lower limbs in humans [2]. However, in children, elderly, and immunocompromised persons, tungiasis may manifest with a severe clinical picture necessitating hospitalization including intensive care [3]. Tungiasis is reportedly endemic in nearly 90 countries; although it does not have a racial or sex predilection, areas of endemicity have poor housing standards, poor sanitation, and poor health literacy.

The diagnosis of tungiasis may be challenging even to an experienced practitioner. Tungiasis may mimic several other clinical conditions including tropical ulcers, acute paronychia, scabies, tick bites, myiasis, warts, granulomas, verruca vulgaris, ecthyma, subungual exostosis, secondary pyoderma, or even early malignant melanoma thus contributing to its misdiagnosis. This diagnostic dilemma is also attributed to the fact that the majority of patients present with already manipulated lesions which may result in secondary bacterial and fungal infections, septicemia, gangrene, or tetanus. We report a case of disseminated tungiasis in a 78-year-old woman of African descent who presented with pain, inflammation, suppuration, ulceration, and deformation of digits in all four limbs.

## Case presentation

**Fig. 1:**
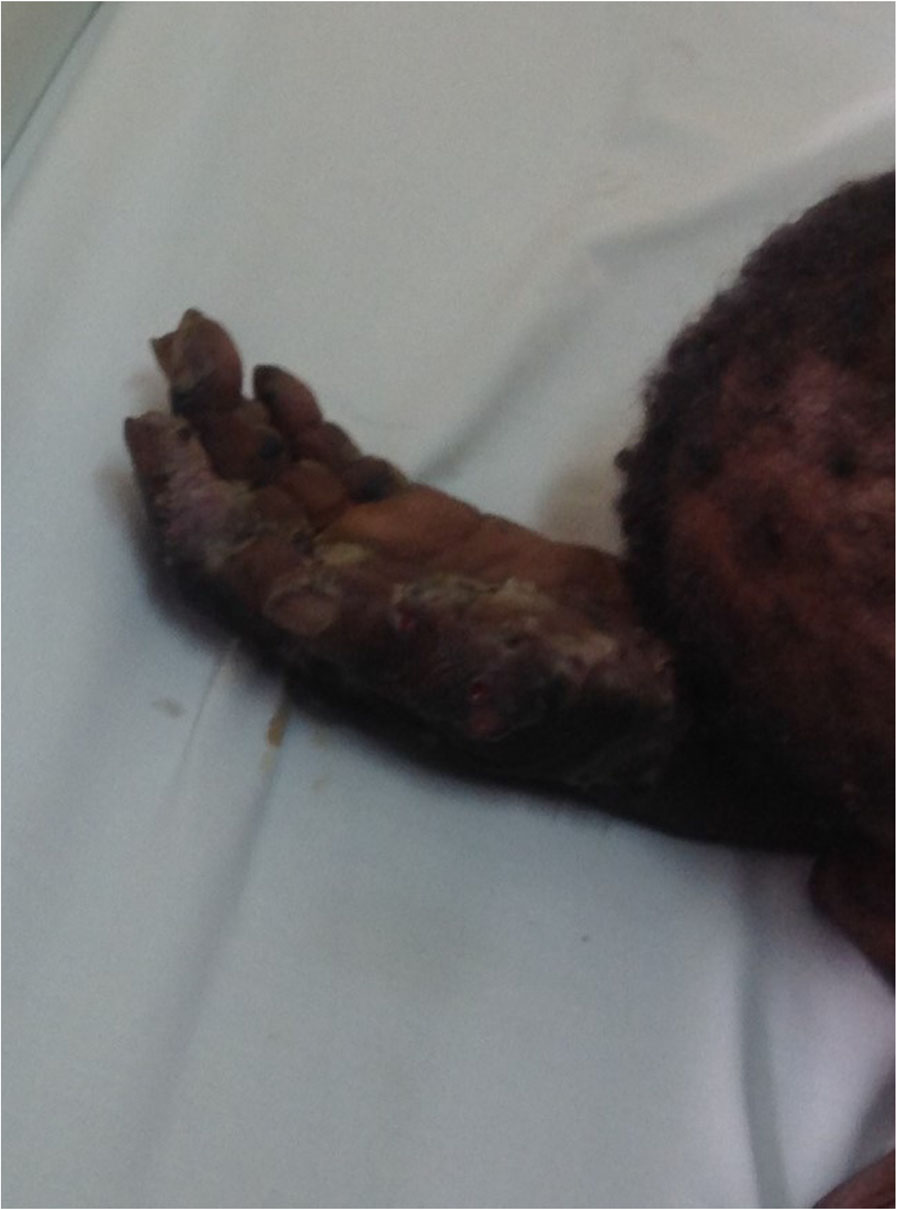
Left upper limb showing ulceration and deformation of digits

**Fig. 2:**
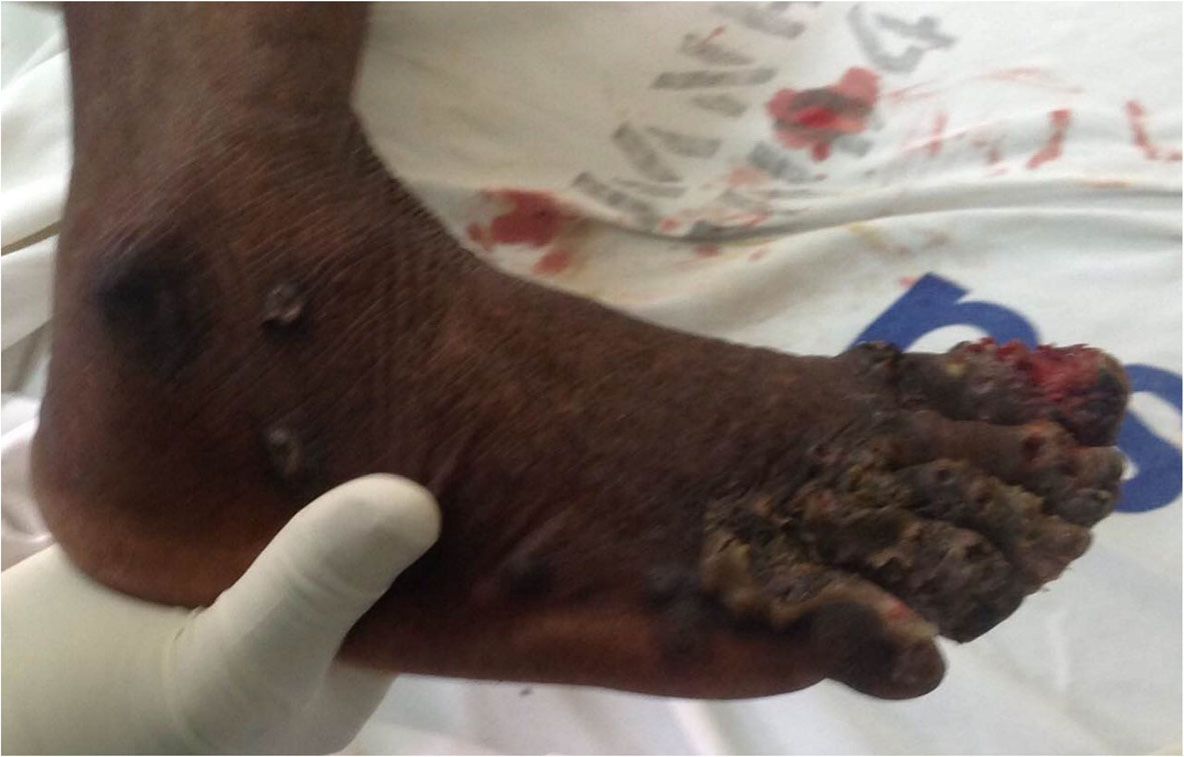
Right lower limb showing ulceration and digit deformation

**Fig. 3:**
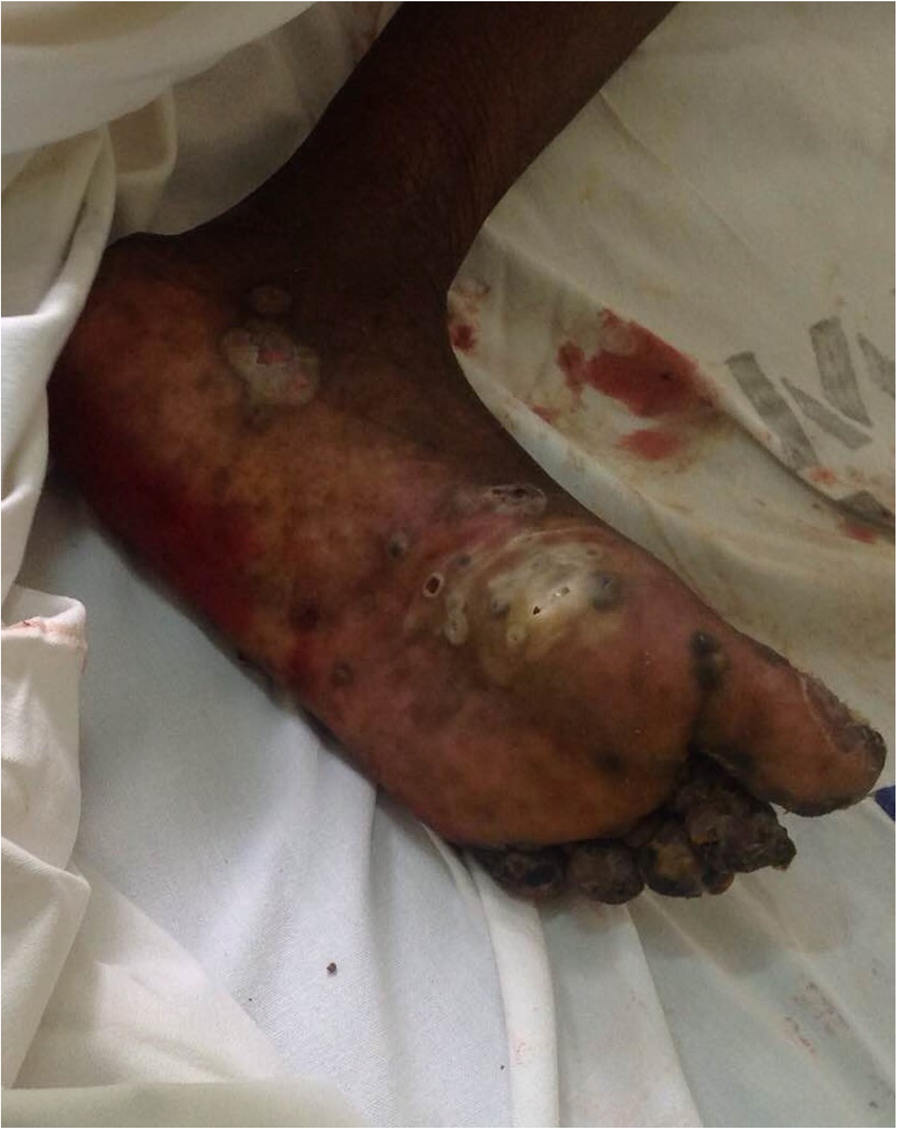
Left lower limb showing sandfly burrows and ulceration

**Fig. 4:**
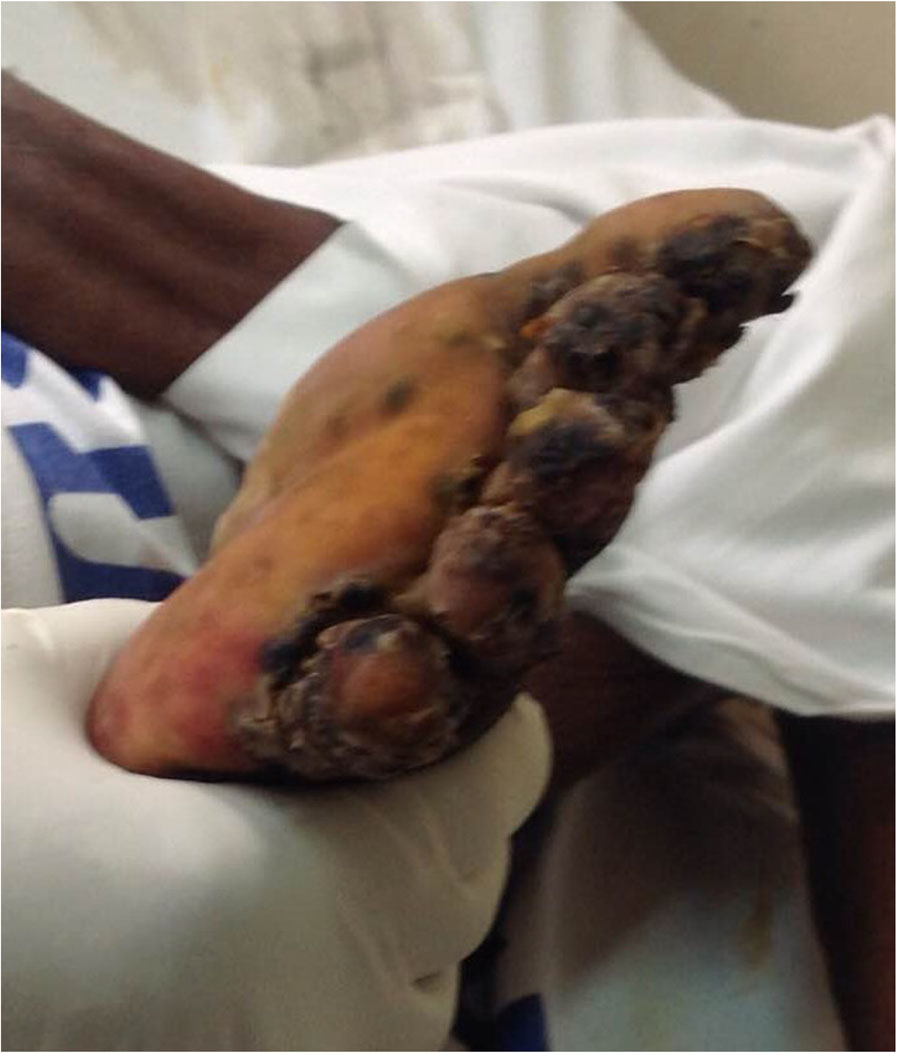
Left lower limb showing severe ulceration and digit deformation

A 78-year-old woman of African descent presented to us with severe inflammation, pain, suppuration, ulceration, and digit deformation in both upper and lower limbs (Figs. 1, 2, 3 and 4). She had a total of 1146 embedded sand fleas: 812 in lower limbs and 334 in her hands. All the nails of her four limbs were lost. She had severe desquamation, persistent pain, and had been unwell for approximately 8 months prior to this index visit. She had manipulated her itchy lesions with unsterile objects and denied a history of over-the-counter medication use. She lived alone in a grass-roofed mud hut with no running water or electricity, and had not taken a shower for a couple of weeks. She had been a peasant all her life but for approximately 1 year she had not taken part in any productive physical activity. Her past medical history could not be ascertained.

She had a blood pressure of 89/54 mmHg, respiratory rate of 28 breaths/minute, heart rate of 108 beats/minute, and temperature of 38.6 °C. She was emaciated with a body mass index of 16.3 kg/m^2^ (weight 44.5 kg and height 1.65 m). A physical examination apart from the obvious skin lesions was positive for conjunctival pallor and reduced muscle bulkiness and power (grade 3/5) in all four limbs. Her full blood picture revealed iron deficiency anemia (hemoglobin, Hb, 7.6 g/dL; mean corpuscular volume, MCV, 62 fL; mean cell hemoglobin, MCH, 19 pg/cell; red cell distribution width, RDW, 23.5%), neutropenia (neutrophils 2.7 × 10^9^/L), and thrombocytopenia (platelets 136 × 10^9^/L). A blood slide for malaria parasite was negative but she had elevated liver enzymes (aspartate aminotransferase, AST, 38 IU/L and alanine transaminase, ALT, 66 IU/L) and hyponatremia (sodium, Na^+^, 116 mEq/L). Her renal functions, bleeding indices, echocardiographic findings, and serology for human immunodeficiency virus (HIV), hepatitis B and C, and Venereal Disease Research Laboratory (VDRL) tests were unremarkable. Blood cultures isolated *Staphylococcus aureus* and *Streptococcus pyogenes* species.

A diagnosis of severe *Tunga* infestation with secondary septicemia was reached based on the typical clinical picture, evidence of embedded sand fleas, and positive blood cultures. We applied 20% salicylated petroleum jelly followed by the manual removal of embedded fleas with a sterile needle. The residual cavity was then cleaned to ensure the entire flea contents were removed. Piperacillin-tazobactam 4.5 g administered intravenously four times a day for 5 days was used successfully to treat sepsis. Moreover, ferrous sulfate (200 mg once a day), folic acid (5 mg once a day), tolvaptan (15 mg once a day), albendazole (400 mg), multivitamins, and tetanus prophylaxis were also administered when her fever subsided. She was also reviewed by nutritionists and she was prescribed a high protein diet. She was discharged home after 16 days of hospitalization with topical ivermectin, ferrous sulfate, folic acid, and multivitamins. She was followed-up through a regional hospital contact at 30 and 90 days with no sign of residual or recurrent manifestation. Furthermore, we managed to contact one of her sons who agreed to live with and take care of his mother at his home located in the city.

## Discussion

Tungiasis remains a neglected yet significant disease associated with considerable morbidity and poor quality of life in endemic areas [3]. In such areas, the prevalence is estimated to range between 15 and 40% [4]. In practice, tungiasis is mostly a clinical diagnosis; however, a definitive diagnosis entails a demonstration of the flea through a mineral oil preparation or skin biopsy [4]. Treatment of tungiasis involves physical removal of the flea by a sharp object followed by cleaning of the residual cavity to remove the remnants. Antibiotic ointment plus a tetanus toxoid immunization following the physical removal has proved to be the most effective treatment strategy [4].

In the case presented, the elderly woman had >1000 lesions in all four limbs and exhibited signs of ulceration, superinfection, and digit deformation. She came from a semi-desert region in central Tanzania which is associated with poor housing and sanitary conditions. Moreover, she faced social neglect for over 6 years following the death of her husband and did not have access to health care. As reported by Feldmeier *et al*. [5], in deprived communities with poor housing standards, social neglect, and insufficient health-seeking behavior, tungiasis has a high transmission potential and increased likelihood for severe disease [5]. A severe form of tungiasis may manifest with digit deformation, chronic lymphedema, sepsis, and tetanus [6]. Our case had severe digit deformation and septicemia. It was unfortunate that our case was actually referred from a regional hospital nearly 600 kilometers away with suspicion of a malignant process. The fact that our case presented to a health facility at such an advanced level of infestation was shocking even to senior practitioners locally. Moreover, the diagnosis challenge caused by the advanced presentation that necessitated such a long referral was indeed a priceless lesson to medical students and junior practitioners at our center, the majority of whom had never seen tungiasis in their lifetime. The authors hope that upon its publication, this case will sensitize practitioners locally and elsewhere regarding the wide spectrum of presentation of tungiasis and its potential for diagnosis confusion.

## Conclusions

In conclusion, tungiasis is a neglected disease of concern in underprivileged societies that is preventable and curable. Early recognition and prompt treatment is crucial to prevent complications in this disease which may potentially mimic other conditions resulting in erroneous treatment.

## Abbreviations

ALT: Alanine transaminase
AST: Aspartate aminotransferase
Hb: Hemoglobin
HIV: Human immunodeficiency virus
MCH: Mean cell hemoglobin
MCV: Mean corpuscular volume
Na^+^: Sodium
RDW: Red cell distribution width
VDRL: Venereal Disease Research Laboratory
